# Correction

**DOI:** 10.1080/15502783.2026.2732598

**Published:** 2026-09-20

**Article title** **:** A 12-Week, Double-Blind, Quasi-Randomized, Placebo-Controlled Study to Evaluate the Efficacy and Safety of Coleus forskohlii (Forcslim) on Body Weight Loss

**Authors** **:** Channangihalli Thimmegowda, S., Hussain Mirza, F. H., Venkataraman, R., & Nagarajaiah, R. B.

**Journal** **:** *Journal of the International Society of Sports Nutrition*

Bibliometrics**:** Volume 23, Number 1, pages 1–17

**DOI:** http://doi.org/10.1080/15502783.2026.2725137

The incorrect figure 1 was originally published online.

The correct figure 1 is now available in the online publication.

